# Skin cancer diagnosis (SCD) using Artificial Neural Network (ANN) and Improved Gray Wolf Optimization (IGWO)

**DOI:** 10.1038/s41598-023-45039-w

**Published:** 2023-11-08

**Authors:** Wanqi Lai, Meixia Kuang, Xiaorou Wang, Parviz Ghafariasl, Mohammad Hosein Sabzalian, Sangkeum Lee

**Affiliations:** 1 The First Clinical Medical School of Guangzhou University of Chinese Medicine, Guangzhou, 510405 Guangdong China; 2 Department of Industrial and Manufacturing Systems Engineering, Kansas State University, Manhattan, KS, 66506 USA; 3 Department of Mechanical Engineering, Faculty of Engineering, University of Santiago of Chile (USACH), Avenida Libertador Bernardo O’Higgins 3363, 9170022 Santiago, Chile; 4 Department of Computer Engineering, Hanbat National University, Daejeon, 34158 Korea

**Keywords:** Cancer, Computer science

## Abstract

Skin Cancer (SC) is one of the most dangerous types of cancer and if not treated in time, it can threaten the patient’s life. With early diagnosis of this disease, treatment methods can be used more effectively and the progression of the disease can be prevented. Machine Learning (ML) techniques can be utilized as a useful and efficient tool for SCD. So far, various methods for automatic SCD based on ML techniques have been presented; However, this research field still requires the application of optimal and efficient models to increase the accuracy of SCD. Therefore, in this article, a new method for SCD using a combination of optimization techniques and Artificial Neural Networks (ANNs) is presented. The proposed method includes four steps: pre-processing, segmentation, feature extraction, and classification. Image segmentation for identifying the lesion region is performed using a Kohonen neural network, where the identified region of interest (ROI) is enhanced using the Greedy Search Algorithm (GSA). The proposed method, uses a Convolutional Neural Network (CNN) for extracting features from ROIs. Also, to classify features, an ANN is used, and by the Improved Gray Wolf Optimization (IGWO) algorithm, the number of neurons and weight vector are adjusted. In this method, a probabilistic model is used to improve the convergence speed of the GWO algorithm. Based on the evaluation results, using the IGWO model to optimize the structure and weight vector of the ANN can be effective in increasing the diagnosis accuracy by at least 5%. The results of implementing the proposed method and comparing its performance with previous methods also show that this method can diagnose SC in the ISIC-2016 and ISIC-2017 databases with an average accuracy of 97.09 and 95.17%, respectively; which improves accuracy by at least 0.5% compared to other methods.

## Introduction

SC is one of the deadliest types of cancer, which in most cases requires surgery^1^. However, in about 20% of cases, even surgery cannot cure this fatal disease. SC alone is responsible for the death of more than 50,000 patients worldwide^1,2^. Unprotected skin exposure to UV rays is the main cause of SC. In most developed countries, SC patients are diagnosed at a very high rate. On the other hand, previous studies show that with early diagnosis of this disease, treatment methods can be applied more effectively and the progress of the disease can be prevented. SC symptoms often show themselves as skin lesions that can only be identified by dermatologists; Because it is very similar to benign lesions. Meanwhile, most of the affected people ignore the skin appendages caused by the disease and do not take any action until the disease progresses. The reason for this can be seen as the difficulty of current diagnosis methods such as sampling. Dermatologists, along with skin sampling as a gold standard; may use several quantitative techniques such as the ABCD rule, 7-point checklist, 3-point checklist^3^, etc. to identify SC in the early stages. But these methods are not very accurate compared to the sampling method. Although it is possible to diagnose SC by processing the images of appendages; however, in diagnosing SC through image processing, we still face challenges such as low accuracy. Therefore, it is necessary to provide methods that can solve these challenges.

Research conducted in recent years has shown that automatic techniques such as machine vision, ML, and deep learning can play an important role in SCD^4^. These capabilities lead to the emergence of various techniques for automatic SCD. However, the relatively low accuracy of these methods shows that we are still far from an ideal automatic SCD system. One of the main reasons for this is the non-use of optimal learning models to detect the type of the lesion and diagnose the disease through it. Although models such as NNs have considerable flexibility and ability to solve classification problems; an efficient diagnosis model based on them requires optimal configuration of NN parameters^5^. Therefore, in this article, an attempt is made to ensure the optimal performance of NNs in SCD by using optimization techniques. The contribution of the current article can be summarized in two cases:

- In this article, an image segmentation algorithm based on the combination of Kohonen neural network and GSA is presented, in which the target regions identified by the Kohonen Model (KM) are improved using GSA.
- In this article, Improved Gray Wolf Optimization (IGWO) is used to configure the structure and weight vector of the ANN. In this way, the number of neurons in the ANN as well as the weight vector between the neurons and its biases are determined using the optimization algorithm. In IGWO, a probabilistic model is used to increase the convergence speed of the optimization algorithm, which can be effective in faster discovering the global optimum of the problem.

The two mentioned cases have not been studied in previous research works for SCD and are innovative aspects of this research. The continuation of this paper includes the following sections: in section “Literature review”, some efforts made for automatic SCD using ML techniques are studied, and in section “Proposed method”, the proposed model is presented. In section “Results”, the results of the proposed method implementation are analyzed, and in section “Conclusion”, the research findings are summarized.

## Literature review

In^6^, an algorithm based on the ABCD rule was introduced for the automatic SCD. In this method, first, the image is pre-processed and the redundant information like hair is removed from the target region, and then segmentation is performed to identify the target region. In the next step, the descriptive features of each extracted region are finally used by the support vector machine (SVM) to classify the features. In^7^, a technique for identifying the lesion region in images related to SC was presented. This method is a solution based on image histogram analysis and uses the color characteristics of the region to identify the lesion region.

In^8^, optimization techniques were used for SCD in images. In this method, first, the input images are pre-processed to remove noise and then the image segmentation is done. In this research, an ANN was utilized for image segmentation, and the weight of its neurons is determined using optimization techniques. In^9^, the combination of a convolutional neural network (CNN) and SVM was employed for SCD. In this research, skin images were used as the input of the CNN, and feature extraction was done by this neural network. This CNN has no classification layer and its last layer creates image descriptive features through a fully connected layer. Finally, the extracted features were used by an SVM and the classification process was done by this learning model.

In^10^, the combination of optimization algorithms and SVM was applied for SCD. In this method, optimization algorithms are used to select the optimal features and SVM is utilized to classify the selected features. In^11^, deep learning techniques were proposed for melanoma diagnosis. In this method, a CNN was used to extract image features. Then these features become the input of two NN models, the first NN is a CNN and was used to identify the target areas. Also, the second NN is a recurrent CNN (RCNN), which identifies the position of the lesion. Finally, based on the determined position, the lesion segmentation was performed using the fuzzy K-means (FKM) algorithm.

In^12^, CNNs were used for SCD and identifying lesion regions. In this model, the input images are pre-processed and then the UNet network is used to segment the images. In the following, the lesion region is cropped based on the result of segmentation and this segment is used as the input of a CNN model called InSiNet to classify the input image. In^13^, an improved CNN model based on the visual geometry group VGG-16 architecture was employed to diagnose SC. In this research, the configuration of the VGG model was modified in such a way that it is more compatible with SCD problems and can be diagnosed with higher accuracy than the basic model. This improvement includes changes in filter dimensions and NN activation functions. In^14^, a method called DUNEScan was introduced, which is a web server for estimating uncertainty in SCD using deep NNs. In this system, several CNN models such as ResNet50T, EfficientNet, Inceptionv3, and MobileNetv2 were used to predict SC, and uncertainty in diagnosis was estimated using the criteria: average, and variance of learning models.

In^15^, the combination of CNN and GWO algorithms was utilized for SCD. In this method, first, the input images were pre-processed so that the redundant information of the images was removed and in addition to that, the CNN training time was improved. Then, the GWO algorithm was used to adjust the hyperparameters of the convolutional neural network. These hyperparameters include the specifications of the convolution filters in the layers of the CNN model. Finally, the best-discovered configuration was used for SCD in new samples.

In^16^, the ensemble model of DNNs was applied for SCD. In this model, the transfer learning process was used and it includes several partial CNN classifications that operate simultaneously in the form of an ensemble system. Finally, an integration model was utilized to combine the results of these models and determine the final output. In^17^, the combination of Fuzzy C-Means (FCM) clustering and the developed red fox optimization (DRFO) algorithm was utilized for SCD. In this method, the input images were segmented through the FCM algorithm to identify the target region. Then feature selection and diagnosis processes were performed. DRFO algorithm was used in both of these steps.

## Proposed method

A method for SCD generally consists of steps for pre-processing, segmentation, feature extraction, and classification. Although some of these steps can be integrated through models such as CNNs. In this section, the details of the proposed method for SCD are provided. The proposed method performs SCD process through the following steps:

1. Preprocessing
2. Segmentation based on the combination of Kohonen neural network and GSA
3. Feature extraction based on CNN
4. Diagnosis based on ANN and IGWO algorithm

**Figure 1:**
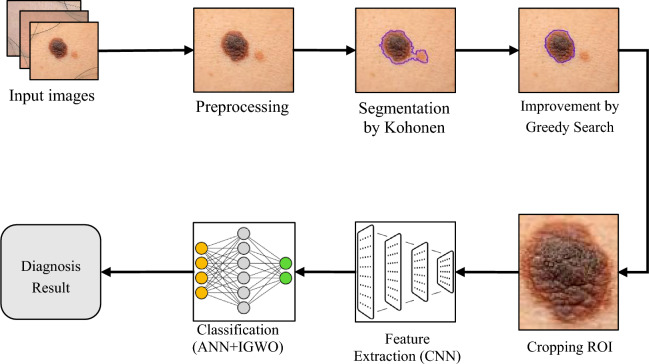
The steps of the proposed method for skin cancer diagnosis.

The steps of the proposed method are represented as a block diagram in Fig. 1. According to this diagram, in the proposed method, first the input images are pre-processed to remove the redundant information in the image such as hair strands or thin blood vessels. Then, each image is segmented using a Kohonen neural network. After segmenting each image, an iterative process based on GSA is used to improve the segmentation regions. The result of this step is a set of segmented images in which the target region is identified as a connected region. In the following, a CNN is used to extract the features of the target region. In this way, the region suspected of melanoma is cropped from the input image and applied to the proposed CNN. This CNN model does not have the necessary layers for classifying features, and the features of each region are described through its last layer. Finally, the combination of ANN and IGWO algorithm is used to classify the extracted features. In this step, the number of ANN neurons as well as the optimal weight vector are adjusted to minimize the training error of the learning model using the IGWO algorithm. In the rest of this section, the details of each of these steps are explained.

### Image preprocessing

The proposed method starts with the pre-processing of the input images. The purpose of the pre-processing step is to remove redundant information and destructive features of the image to improve the performance of the SCD system in the next steps. The recorded images of the skin face two major destructive factors:

- Destructive effects caused by imaging that appear as a blur, noise, or light reflection in the skin image.
- Hair strands and blood vessels in the skin that can cause errors in the diagnosis process.

In the pre-processing step of the proposed method, to remove the effect caused by the mentioned factors, the morphological closing operator and blur removal by the unsharp filter^18^ are utilized. In this way, it is first tried to remove the blood vessels and hair strands using the morphological closing operator. This operator can be defined as (1).

1 $$\hat{I}_{x,y} = \left( {I_{x,y} \oplus C_{1} } \right){ \ominus }C_{2}$$

where ${I}_{x,y}$ is the initial input image and ${\widehat{I}}_{x,y}$ is the image resulting from applying the morphology operator. Also, ${C}_{1}$ and ${C}_{2}$ respectively represent 10 pixels in the directions of 90 and 180 degrees against each other pixel. By applying this operator to the input image, the blood vessels and hair strands in the image (due to their low thickness which is less than 10 pixels) are removed. But this process causes blur in the image ${\widehat{I}}_{x,y}$. Therefore, the unsharp filter is used to remove image blur. This action can be formulated as (2)^18^.

2 $$\overline{I}_{x,y} = \hat{I}_{x,y} \times \left( { - \frac{1}{{\pi \delta^{4} }}\left[ {1 - \frac{{x^{2} + y^{2} }}{{2\delta^{2} }}} \right]e^{{\left( {\frac{{x^{2} + y^{2} }}{{2\delta^{2} }}} \right)}} } \right)$$

where x and y represent the length and width of the image, respectively. Also ${\overline{I} }_{x,y}$ is the result of applying the unsharp filter on the image ${\widehat{I}}_{x,y}$, which can be used to improve the quality of the image. For this purpose, using Eq. (3) the image ${\overline{I} }_{x,y}$ is subtracted from the input image ${I}_{x,y}$ so that the result of the pre-processing step is created as a high-resolution image free of arteries and hair strands:

3 $$P_{x,y} = I_{x,y} - \overline{I}_{x,y}$$

where, ${P}_{x,y}$ represents the results of preprocessing image ${I}_{x,y}$. The set of images resulting from the above steps is used as the input of the second step of the proposed method so that segmentation and diagnosis of disease can be done based on them.

### Image segmentation based on Kohonen neural network and GSA

The second step of the proposed method is to segment the pre-processed images and identify the target region, which is done using the combination of Kohonen neural network and GSA. We consider an image such as ${I}_{x,y}$ where $x\in [1,{N}_{x}]$ and $y\in [1,{N}_{y}]$ determine the position of each pixel and $i(x,y)$ is the intensity of the pixel with the specified position. The image segmentation problem ${I}_{x,y}$ is finding meaningful subsets of the image so that the conditions of Eq. (4) can be met:

4 $$\mathop \cup \limits_{k = 1}^{N} S_{k} = I$$

$$\forall k,j \in \left[ {1,N} \right], k \ne j| S_{k} \cap S_{j} = \emptyset$$

where *S*_*k*_ is the *k*th segment of the image and *N* is the total number of formed regions. The first relation in (4) specifies that the sum of all the formed regions must reconstruct the entire image *I*; while the second relation expresses that the regions do not have common pixels. Each image in the segmentation problem can be defined as a matrix. Therefore, the image segmentation problem converts to a numerical matrix clustering problem, which is done using the Kohonen neural network. This learning model can be used for clustering or classification purposes. In the proposed method, the clustering capability of this model is utilized. Using Kohonen learning rule for data clustering leads to the formation of a competitive and unsupervised learning model.

Kohonen neural networks only include two layers: the input layer and the competitive output layer. Each neuron in the input layer is connected to competing neurons. Meanwhile, each neuron in the competitive layer may be connected to all other competitive neurons. A network with Kohonen learning rule is a type of network in which the neurons are configured as a two-dimensional layer with defined topology. Defined topology means that for each neuron there is a predetermined set of neighboring neurons. By applying the input vector to the network weight vector, the output of the network is determined. “Similarity” is used as a criterion to determine the winning neuron in this network. In other words, there is a competition to win between neurons in this network. In the proposed method, the minimum Euclidean distance is used as a measure of similarity in the Kohonen neural network.

Kohonen learning rule tries to map an input vector to a column of neurons that are more similar to the input vector. We consider a Kohonen neural network, each layer of which is a set of neurons forming a two-dimensional structure. The number of layers in this network is equal to the length of the input feature vector (number of image pixels). Upon receiving the input, the winning neuron is determined based on the similarity between the two sets, and the weight of each neuron is updated to minimize the difference between the target output and the output of the neuron. In the learning phase of this model; Each neuron calculates the distance between the input vector and its weights. The neuron with the closest weight to the input vector is the winner of this competition stage, for which the corresponding *Z*_*i*_ is set equal to 1 and the other *Z*_*i*_ s are equal to zero. Then Kohonen rule is used to update the weights as (5):

5 $$w_{i,j}^{new} = \left\{ {\begin{array}{*{20}l} {\left( {1 - \alpha } \right)w_{i,j}^{old} + \alpha .x \,\,for\,\, winner} \\ {w_{i.j}^{old} \,\,other \,\,unites} \\ \end{array} } \right.$$

The pseudocode of the Kohonen neural network training algorithm in the proposed method is as follows^19^.

**Figure:**
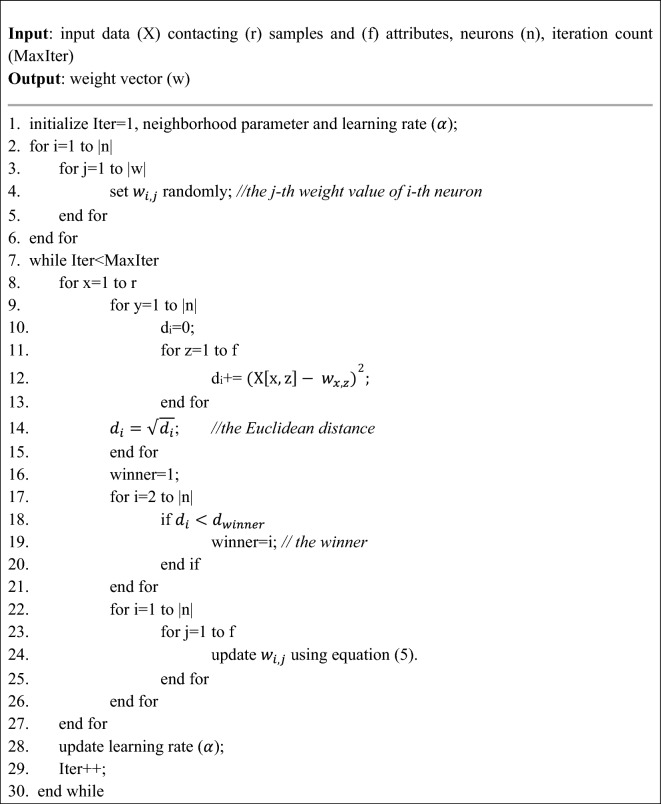
Algorithm 1 Kohonen neural network training

During this process, the learning rate $\alpha$ can decrease linearly during training. Also, for the algorithm to converge, it may be necessary to apply the training set to the network many times. In the simplest case, the output of an NN is the weighted sum of the inputs, which is usually calculated through a sigmoid function. If this state is considered, the output is a single 1 in a region of the coordinate space that corresponds to a specific class of patterns.

After generating the output of Kohonen neural network, GSA is used to improve and optimize the regions determined in the image. In order to use GSA in the proposed method, first a segmenting matrix such as $u$ is extracted from the output of Kohonen neural network ($S$) and during an iterative process, the improvement of the regions determined by $u$ is performed. The $u$ matrix is defined as a binary matrix of the same size as the segmentation matrix $S$, in which the outer connected region is assigned 0 and the inner connected region/regions, is/are assigned 1. Thus, in the $u$ matrix, regions specified with 0 represent the background of the image, and regions specified with 1 are the initial approximation of the target region. In each GSA iteration, first, the average intensity of the current region's pixels (in the pre-processed image) is calculated and added to the coefficients matrix of the KM. In the resulting matrix, which is called $K$, the minimum Euclidean distance between the coefficients is calculated. The coefficients that have the smallest distance in this matrix are called ${I}_{in}$. The same operation is done for the average intensity of the pixels outside the region, and the coefficients that have the smallest distance in this matrix are called ${I}_{out}$. Then, having the horizontal and vertical gradients of $u$ as $G_{x}$ and $G_{y}$, $u$ is updated as (6):

6 $$u^{\prime} = u + \left[ {\left( {X - K\left( {I_{out} } \right)} \right)^{2} - \left( {X - K\left( {I_{in} } \right)} \right)^{2} } \right] \times \sqrt {G_{x}^{2} + G_{y}^{2} }$$

This process is repeated until $u\ne {u}{\prime}$ at the end of the iteration. After the end of this iteration, the final ${u}{\prime}$ is the image segmentation matrix, and the positive values of the matrix are the pixels inside the target region and the other pixels outside the target region. In the following, this area is cropped and used as input for the next step.

### Feature extraction based on CNN

**Figure 2:**
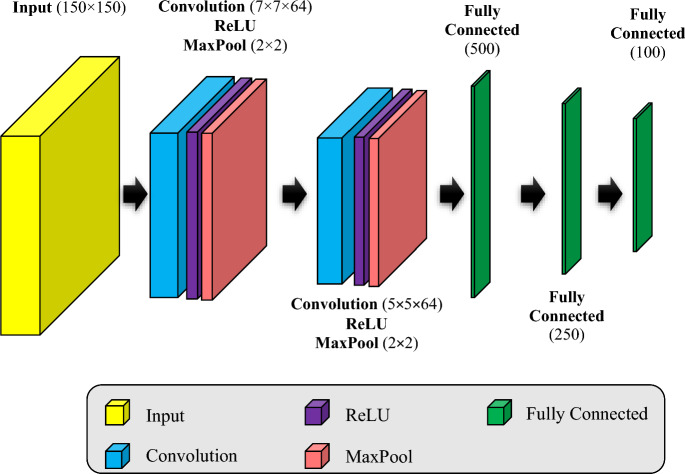
The proposed CNN model for image feature extraction.

In the third step of the proposed method, the features of the target region are extracted using a CNN. The structure of the CNN used in the proposed method is shown in Fig. 2. According to this figure, the proposed CNN's input is the cropped images of the target area. Considering that the same dimensions of the input samples is one of the design requirements of CNN models; Therefore, at the beginning of this step, the image dimensions of the target region are changed to 150 × 150 pixels. The input image is processed through two consecutive convolution layers to extract the patterns in the texture of the images using the filters of these two layers. The first convolution layer has dimensions of 7 × 7 and consists of 64 filters. While the second convolution layer has 64 filters with dimensions of 5 × 5. The number and dimensions of filters in each convolution layer are determined experimentally and based on the conditions of the samples. Based on the experiments, the use of fewer filters leads to a decrease in detection accuracy; while increasing the number of filters more than 64 leads to over-fitting of the model. In this case, the performance of the model in the test phase is not suitable. In this architecture, the first convolution layer is responsible for extracting the partial patterns of the image; while the extraction of global patterns is done through the second convolution layer. Each convolution layer is followed by a ReLU layer and a MaxPool layer. In this case, the ReLU layer acts as an activation function; While the MaxPool layer is responsible for sampling the data and transferring it to the next layers. After extracting the patterns by these two layers, the features of the input image are extracted through three fully connected layers. The first fully connected layer defines the features of the image with a vector of length 500, and then the second and third fully connected layers reduce the dimensions of these features to 250 and 100 features, respectively. Based on the experimental tests, the gradual reduction of features using these three fully connected layers in the proposed CNN model can prevent the loss of useful features in skin cancer diagnosis. In this way, this architecture can increase the accuracy of detection and the weight values obtained for each image through the third fully connected layer specify the characteristics of that image and are used as the input of the classification model.

### Classification of features based on ANN and IGWO

**Figure 3:**
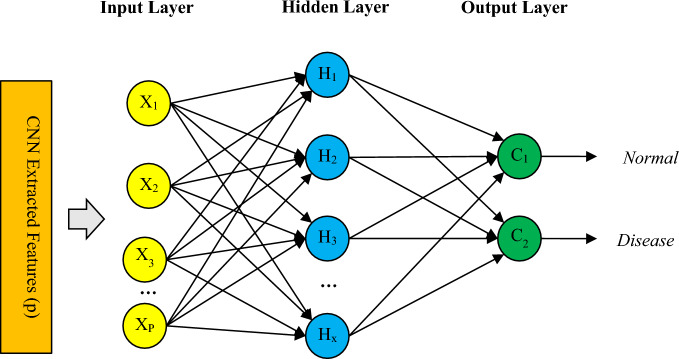
The proposed NN structure for disease diagnosis.

The last step of the proposed method is the classification of features that were extracted by CNN model. Although this operation can be performed by adding SoftMax and Classification layers to the end of the CNN model, but this structure cannot guarantee the achievement of high diagnosis accuracy. For this reason, in the proposed method, feature extraction and classification processes are separated so that the diagnosis of the disease can be done with higher accuracy. therefore, in the fourth step of the proposed method, an ANN is used to classify the extracted features. The structure of this NN is depicted in Fig. 3. Based on Fig. 3, the number of input neurons of the proposed ANN model is equal to the number of features extracted through the CNN model. Also, this NN includes two output neurons that separate healthy and diseased categories respectively. In this NN, one hidden layer is considered which uses tangent sigmoid (tansig) as its transfer function. The requirement to achieve the highest classification accuracy by this ANN model is to determine the optimal solution for two interdependent problems: First, the optimal structure for this ANN model must be determined in the form of the number of neurons in its hidden layer. Using a small number of neurons can lead to low diagnosis accuracy; While using a large number of neurons leads to overfitting in the ANN training process. Secondly, the bias values and the weight vector of NN neurons should be determined in such a way as to cause the least diagnosis error.

The performance of common algorithms for training NNs is highly dependent on the structure of the network and the input data, and for this reason, it cannot be sure that these methods have similar performance in real-world conditions. In the proposed method, the GWO algorithm is used to solve these two problems. Thus, in this step, an attempt is made to determine the number of optimal neurons as well as the weight vector of the NN using the GWO algorithm. In the following, first, the structure of the solution vector and the fitness function in the optimization problem of this research are presented, and then the steps to solve this problem using the GWO algorithm are described.

In optimization algorithms, each solution vector represents a possible solution to the problem. In the proposed method, the following variables are optimized using each solution vector:

- The number of neurons in the hidden layer of the NN: in this case, the optimal structure of the NN is determined based on the size of its hidden layer. For this purpose, the first optimization variable in each solution vector represents the number of neurons in the hidden layer, which is determined as a natural number in [5,30].
- The weight values between neurons and biases of the NN: Determining the appropriate values for these optimization variables means training the NN. Thus, by using the optimization algorithm, in addition to the configuration of the NN, its training is also performed. The length of the weight vector in an NN depends on the number of its neurons. Therefore, the number of this group of optimization variables depends on the first variable (the number of hidden neurons). In an NN with X input neuron, H hidden neuron, and C output neuron, the weight vector length is equal to $H\times \left(X+1\right)+C\times \left(H+1\right)$. Each solution vector in the proposed method determines the weight values as a real number in the interval [− 2, + 2].

In this way, the solution vectors in the proposed optimization algorithm have variable lengths, in which the first part specifies the number of neurons in the hidden layer of the NN and the other part determine the weight vector of that network.

After determining each solution vector, it should be possible to calculate its fitness using a suitable criterion. In the proposed method, the training error criterion is used as the fitness function. With these explanations, to evaluate the fitness of each solution, first, the NN is configured according to the number of neurons and weight values determined in the solution vector. Then the training samples are applied to this NN and the output of the NN is extracted for these samples. Finally, the training error is calculated using Eq. (7) by comparing the output of the NN and the actual labels of the training samples:

7 $$Fit = \frac{F}{N}$$

where *F* is the number of training samples that have inconsistent output with real labels, and *N* shows the total number of training samples. Thus, in the proposed method, the Improved GWO algorithm, searches for a configuration of ANN that can minimize training error (Eq. 7). The GWO algorithm is an optimization strategy based on the collective behavior of the wolf pack in hunting prey. In this algorithm, the best solutions in the first, second, and third ranks are known as α, β, and δ, respectively, and other members of the wolf pack are displayed as a set of ω. Each member of the set ω follows members α, β, and δ to search for prey, and surrounds the prey when it is discovered. This process is simulated in the GWO algorithm with (8)^20^.

8 $$P_{t + 1} = L_{t} + A \times D$$

where $L_{t}$ represents the location of the prey and *t* represents the number of iterations of the algorithm. The parameter $P_{t + 1}$ indicates the position of the gray wolf in the next iteration cycle. Also, A and D are the prey encircling coefficient vectors that are calculated as (9) and (10)^20^:

9 $$A = 2A \times r_{1} - a$$

10 $$D = \left| {2r_{2} \times L_{t} - P_{t} } \right|$$

where, $r_{1}$ and $r_{2}$ are random parameters in [0, 1]. The elements of the vector $a$ decrease from 2 to zero during the iterations of the optimization algorithm and is updated based on (11)^20^:

11 $$a = 2 - \frac{2t}{G}$$

where *G* represents the maximum number of iterations of the optimization algorithm. In the natural behavior of the gray wolf herd, members of the herd follow *α* and in some cases follow *β* and *δ* to hunt. The basic model of the GWO algorithm also uses the same process. But it is possible to use a larger set of appropriate solution vectors discovered during previous iterations to generate new solutions. In the proposed method, a list *S* is used to store the previous best solutions. In addition to this set, vector *Z* is used to determine the probability of selecting each member of *S* in determining the position of gray wolves. The purpose of using these sets in the proposed method is to increase the probability of discovering the global optimal solution by searching around some top solutions. In this case, during each iteration of the optimization algorithm, the top three solutions in the list *S* are searched. If any of these solution vectors are not available in the set *S*, and its fitness is lower than the average fitness of the set *S*, then the mentioned solution vector is added to the set *S*. After adding each new member to the set *S*, the probability vector *Z* is updated as (12):

12 $$z_{i} = \frac{{1 - Fit\left( {s_{i} } \right)}}{{\mathop \sum \nolimits_{j = 1}^{\left| S \right|} \left( {1 - Fit\left( {s_{j} } \right)} \right)}}$$

In the above equation, $Fit\left( {s_{i} } \right)$ represents the fitness of the *i*-th member of the set *S* and is calculated based on (7). Also |*S*| represents the number of members of the set *S*. During each iteration of the optimization algorithm, half of the gray wolf population members determine their position based on *α*, *β*, and *δ* members; while the position of the second half of the population is determined based on the three members of the *S* set. In this case, three members of this set are selected based on the roulette wheel algorithm. This process improves the search process in the problem space for the GWO algorithm and therefore we call it IGWO. With these explanations, the steps of the proposed IGWO algorithm for optimizing the structure of the ANN include the following steps:

1. Determine the initial population randomly and value the vectors of initial coefficients.
2. Compute the fitness of the solution vectors in the population and update the members *α*, *β*, *δ*, and *ω*.
3. Update the members of the set *S* and the probability vector *Z*.
4. For half of the population of wolves, execute the following commands:
  - Select three members of the set *S* based on the roulette wheel algorithm.
  - Determine the new position of the wolf based on the selected members of *S*.

5. In the remaining half of the population of wolves, use members *α*, *β*, *δ* to determine the new position of each wolf.
6. Calculate the fitness of each solution vector.
7. Update vectors *A,* and *a*.
8. Check the termination conditions and if the conditions are not met, repeat the algorithm from step 2.

In the IGWO optimization algorithm, the termination conditions are:

- The number of iterations of the algorithm reaches the predetermined threshold *T*.
- The fitness of the best-discovered solution is zero.
- The fitness of the best-discovered solution does not improve after *m* iterations.

After determining the best solution by the proposed IGWO algorithm, the ANN is configured based on it and used to classify new samples.

## Results

The proposed method was implemented using MATLAB 2019a software. During the evaluations, the performance of the proposed method has been studied using the precision, accuracy, recall and F-Measure criteria, and the results have been compared with previous similar studies. In this research, the samples of two databases ISIC-2016^21^ and ISIC-2017^22^ were used. At the beginning of this section, the specifications of the used databases and the implementation parameters are described, and then the evaluation results based on these two databases are analyzed.

Both ISIC-2016 and ISIC-2017 databases are provided by International Symposium on biomedical images (ISBI) in line with the challenge of skin lesion analysis for skin cancer diagnosis. The ISIC-2016 database consists of two parts, training and test samples. The number of training samples of this database is equal to 900 color images. Also, in this database, the number of test samples is 379, in which 304 samples are in the normal class and 75 samples are in the melanoma class. On the other hand, the ISIC-2017 database consists of more samples. This database includes 2000 training samples and 600 test samples. In the test set of this database, there are 483 normal samples and 117 samples with melanoma. The samples of these two databases have effects such as blur, reflection, noise, as well as the presence of hair strands and thin veins, which makes the diagnosis process challenging. It should be noted that at the time of writing this article, the ISIC database has three newer databases, ISIC-2018, ISIC-2019 and ISIC-2020, which, due to the large volume of samples in this database, cannot be used in the testing process.

In the implementation method, the collection of training samples of these databases was used to form the proposed model, and then its performance was evaluated using test samples. In order to evaluate the effectiveness of the proposed method, criteria of precision, accuracy, recall and F-Measure were used. After classifying the test samples by the proposed model (or other compared models), the predicted class for each of the test samples is compared with the actual label of that sample. In this case, a test sample can be placed in one of the following classes:

- TP or true positive, which indicates the number of melanoma samples that are correctly classified.
- TN or true negative, which shows the number of non-melanoma samples for which the prediction is made correctly.
- FP or false positive, which indicates the number of test samples without melanoma, which is incorrectly classified as melanoma.
- FN or false negative, which specifies the number of melanoma samples that are incorrectly classified as non-melanoma.

The accuracy criterion indicates the number of positive or negative test samples that the proposed detection model was able to classify correctly. The precision criterion shows what proportion of the positive outputs of the classification algorithm is correct. On the other hand, the recall criterion determines the proportion of correctly classified positive samples. These criteria are calculated using Eqs. (13–16):

13 $$Accuracy = \frac{TP + TN}{{TP + TN + FP + FN}}$$

14 $$Precision = \frac{TP}{{TP + FP}}$$

15 $$Recall = \frac{TP}{{TP + FN}}$$

16 $$F - Measure = \frac{2 \times Precision \times Recall}{{Precision + Recall}}$$

In addition to these criteria, Classification Success Index (CSI) and Matthews correlation coefficient (MCC) criteria were also used for better studying the performance of the proposed method. The CSI and MCC criteria can be formulated as (17) and (18), respectively:

17 $$CSI = PPV + TPR - 1 = \frac{TP}{{TP + FP}} + \frac{TP}{{TP + FN}} - 1$$

18 $$MCC = \frac{TP \times TN - FP \times FN}{{\sqrt {\left( {TP + FP} \right) \times \left( {TP + FN} \right) \times \left( {TN + FP} \right) \times TN + FN)} }}$$

The CSI in Eq. (17) describes how successful a classifier operates in correctly diagnosing. The MCC criteria in Eq. (18) rates the performance of diagnosis system in classifying (positive and negative) samples. In the worst case, the MCC would be -1 while the best case is identified by MCC = 1.

**Figure 4:**
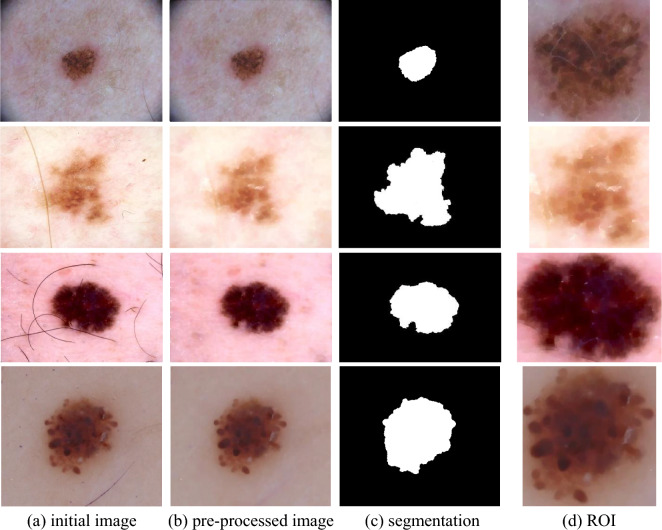
Pre-processing, segmentation, and ROI extraction for some samples.

As mentioned in the previous section, in the proposed method, the combination of the Kohonen neural network and greedy learning strategy is used to segment the input images and identify the target region. Figure 4 shows the image segmentation results of ISIC-2016 and ISIC-2017 database samples. In this figure, each line specifies the processing steps of an image sample. Also, in each column, one of the primary processing steps of the proposed method is displayed. The first column specifies the initial input images. In the second column, the pre-processing result is displayed. The third column shows the result of segmentation by Kohonen neural network improved by GSA. Finally, the target region extracted from the image is plotted in the fourth column.

In Fig. 4, the two images displayed in the first and second rows belong to the ISIC-2016 database, and the other two images are extracted from the ISIC-2017 database.

**Figure 5:**
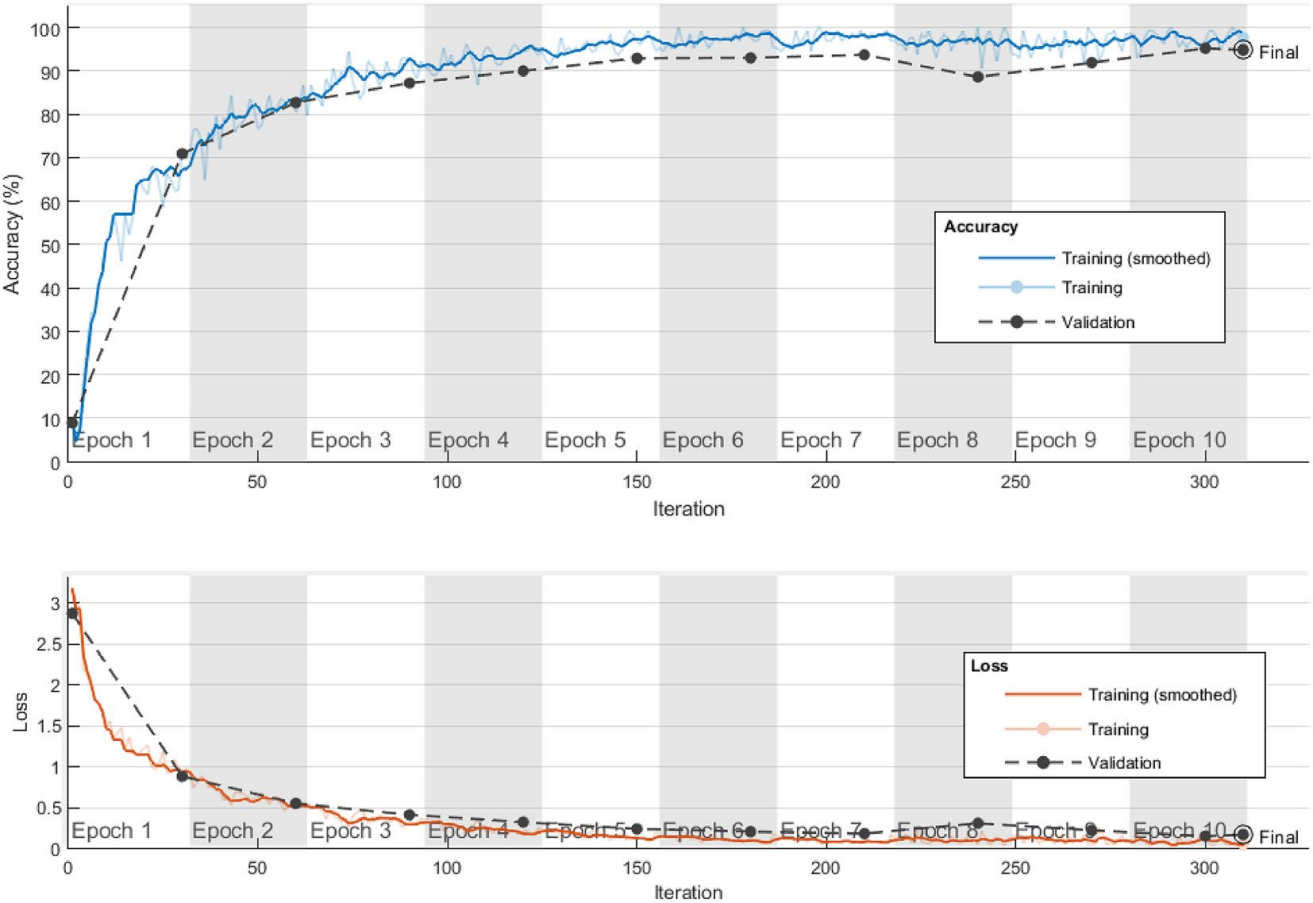
Accuracy and cross entropy loss of proposed CNN during training.

As the images in Fig. 4 show, the proposed method can eliminate the adverse effects caused by hair strands and thin blood vessels during the pre-processing process. On the other hand, the segmentation step of the proposed method has acceptable performance and can detect the target region with high accuracy. The detected ROIs were fed to the proposed CNN model for feature extraction. Figure 5, illustrates the accuracy and cross entropy loss of proposed CNN during training phase.

**Figure 6:**
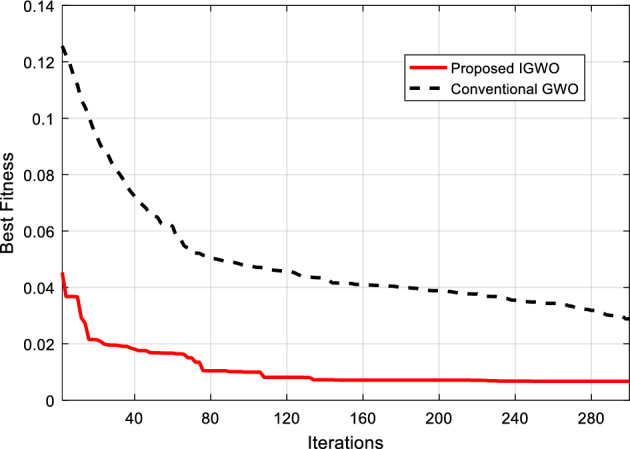
Comparing the variation of best fitness during ANN configuration by IGWO and GWO.

According to the procedure described in section “Classification of features based on ANN and IGWO”, the IGWO algorithm was used to configure and determine the weight vector of the ANN. In the process of implementing the IGWO algorithm, the parameters of the number of iterations and the population size were determined to be 300 and 250, respectively. Also, the threshold of the number of iterations without improvement in the best fitness, which is considered one of the termination conditions, was set equal to m = 100. In Fig. 6, the graph of the changes in the fitness of the proposed IGWO algorithm in the optimal configuration of the ANN is displayed. In this figure, the results of the proposed IGWO algorithm have been compared with the case where the basic GWO algorithm was used to optimize the NN model.

The comparison of the proposed IGWO algorithm with the basic GWO method shows that the proposed method can achieve solutions with a better fitness in the same number of iterations. On the other hand, the fitness of the best solution discovered by the proposed method after 300 iterations of the optimization algorithm is lower than the compared method. Based on these results, the proposed method could determine the optimal configuration for the NN and its training with a training error rate of less than 0.007. This is while the training error of the ANN model optimized by the GWO algorithm is 0.029. Thus, in addition to faster convergence, the proposed IGWO algorithm can achieve more suitable solutions with the same number of iterations.

In the following, the results of the classification of the images of the ISIC-2016 and ISIC-2017 databases are analyzed. In these tests, to evaluate the effectiveness of the techniques used in the proposed method, the results have been compared with the following situations:

- Classification of features based on ANN with Levenberg–Marquardt (LM) training function. This NN has 12 neurons in its hidden layer.
- Classification of features based on ANN model optimized by basic GWO. In this case, the parameters of the optimization algorithm are similar to the values set for the proposed method.
- Classification of features by the proposed CNN. In this case, a sequence of SoftMax and classification layers are added to the end of proposed CNN model and the extracted features are classified by these layers. In other works, this case refers to the scenario that SCD by combination of ANN and IGWO is ignored.

**Figure 7:**
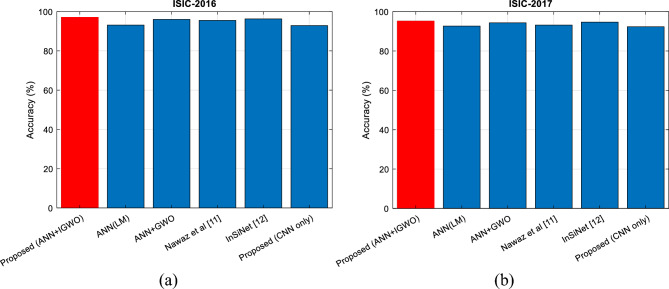
Accuracy of SCD in: (**a**) ISIC-2016 and, (**b**) ISIC-2017 databases for different methods.

Also, in these tests, in addition to the above situations, the performance of the proposed method has been compared with the methods presented in^11^ and^13^. SCD was done using test samples from two databases ISIC-2016 and ISIC-2017. Based on the test results, the proposed method could classify the test samples of the ISIC-2016 database with 97.09% accuracy. On the other hand, for the samples of the ISIC-2017 database, this accuracy is 95.17%. The accuracy results of all tested algorithms are shown in Fig. 7.

Based on Fig. 7, the proposed method could achieve higher detection accuracy than the compared methods for both ISIC-2016 and ISIC-2017 databases. Based on these results, in the evaluation by the samples of the ISIC-2016 database, the proposed method can increase the accuracy by at least 0.79 percent, and this improvement rate for the ISIC-2017 database is at least 0.5 percent, which in both these cases, the InSiNet method has the closest performance to the proposed method.

On the other hand, the comparison of the proposed classification model (combination of ANN and IGWO) with the ANN based on the LM training function shows that the proposed optimization strategy can increase the diagnosis accuracy by 2.5% compared to CNN models. This difference in classification accuracy shows the effectiveness of the proposed optimization strategy for adjusting the number of neurons and the weight vector of the NN. To check the effectiveness of the IGWO algorithm proposed in this article, it is possible to compare the accuracy of the proposed method with the ANN + GWO mode in which the basic GWO algorithm is used for NN optimization. Based on these results, the proposed method in both ISIC-2016 and ISIC-2017 databases can configure the ANN model more efficiently; in such a way that the accuracy of SCD in database samples increases by about one percent.

**Figure 8:**
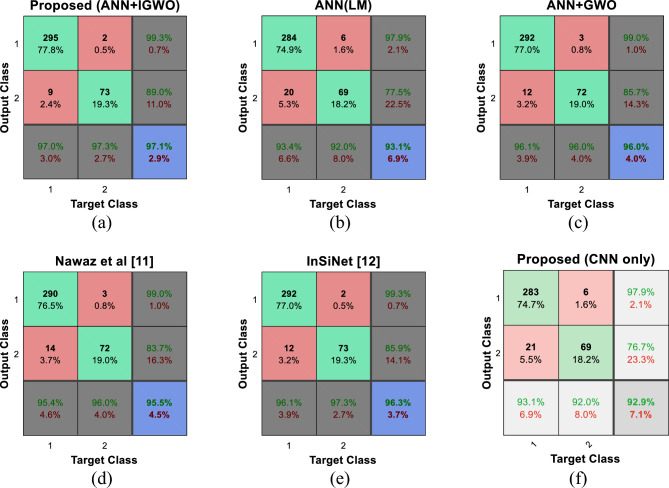
Confusion matrix resulting from SCD in ISIC-2016 database by different methods.

Figure 8 shows the confusion matrix of different algorithms for classifying test samples of the ISIC-2016 database. Figure 8a shows the confusion matrix of the proposed method. In these matrices, labels 1 and 2 represent classes of non-melanoma and melanoma, respectively. Also, the rows of these matrices represent the labels predicted by each method; while the columns of the matrix represent the ground-truth labels. In this way, the proposed method has correctly classified 295 images of the test samples of the non-melanoma category (TN) and 9 test samples of this class have been wrongly diagnosed as melanoma (FP). On the other hand, out of 75 test samples with melanoma, 73 images were correctly classified (TP) and the remaining two images were wrongly diagnosed as non-melanoma (FN). In this way, the proposed method only had an error in the classification of 11 samples out of 379 test samples of the ISIC-2016 database, which resulted in 97.09% accuracy of the proposed method. Based on these results, the performance of the proposed method is 97.3 and 97% in terms of sensitivity and specificity criteria, respectively.

Comparing the confusion matrix of the proposed method with other methods shows that the method proposed in this research has a better performance for SCD from the samples of the ISIC-2016 database. Based on these results, the InSiNet method has the closest performance to the proposed method and has been able to correctly classify 96.3 samples. Based on these results, the sensitivity and specificity of this method are 97.3% and 96.1%, respectively.

**Figure 9:**
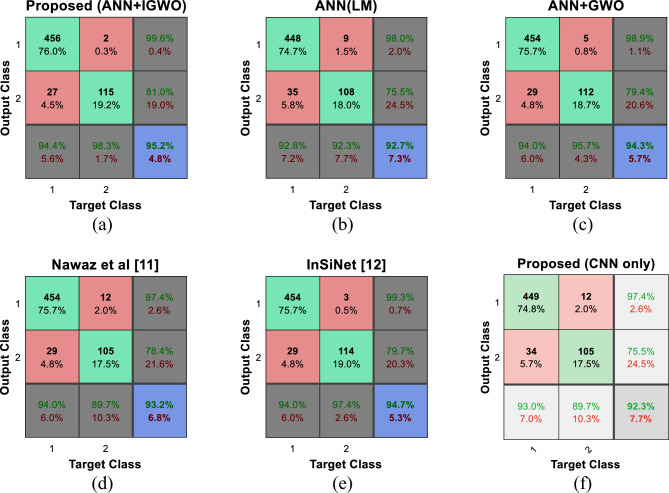
Confusion matrix resulting from SCD in ISIC-2017 database by different methods.

The confusion matrix of different algorithms for the classification of ISIC-2017 database test samples is shown in Fig. 9.

The comparison of the matrices shown in Fig. 9 shows that the proposed method has a better performance than the compared methods in the ISIC-2017 database. Therefore, the sensitivity and specificity of the proposed method after SCD in the test samples of this database are 98.3 and 94.4%, respectively, which compared to the closest method (InSiNet) has been able to meet these criteria by 0.9% and increase by 0.4 percent. In this way, the proposed method is superior to the compared methods in the correct classification of both non-melanoma and melanoma samples.

**Figure 10:**
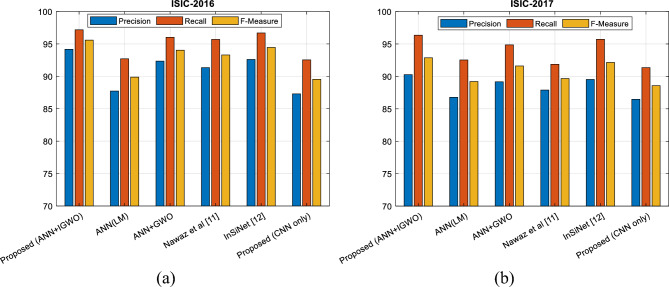
Classification quality of SCD for (**a**) ISIC-2016 and (**b**) ISIC-2017 databases by different methods.

In addition to the sensitivity and specificity criteria, confusion matrices can also be used to evaluate precision, recall, and F-Measure criteria. These criteria are calculated based on Eqs. (14–16). In Fig. 10, different SCD methods are compared in terms of precision, recall, and F-Measure for two databases ISIC-2016 and ISIC-2017.

**Figure 11:**
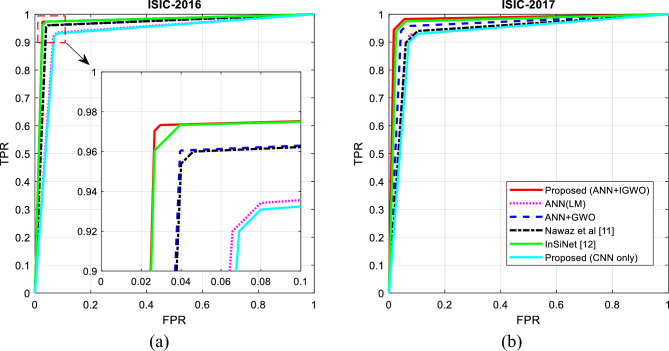
ROC curve resulting from the classification of database samples: (**a**) ISIC-2016, (**b**) ISIC-2017 by different methods.

The results presented in Fig. 10, confirm that the proposed method can diagnose SC in the used databases with higher efficiency. The higher precision criterion of the proposed method means that the outputs classified in the melanoma class by the proposed method have a higher probability of correctness. On the other hand, the higher recall criterion shows that the proposed method can correctly classify a higher rate of melanoma samples. The superior performance of the proposed method can be seen as the result of using the IGWO algorithm in optimizing the configuration of the ANN model. This optimization algorithm tries to adjust the number of hidden neurons and the weight vector of the NN in such a way that it achieves the lowest possible error. On the other hand, using the history list of the best solutions and the probabilistic model used in the proposed IGWO algorithm makes it converge faster to the global optimal solution. Figure 11 shows the ROC curve resulting from the classification of samples from two databases used for different methods.

In Fig. 11, the changes of the true positive rate (TPR) for different false positive rate (FPR) thresholds are displayed. Based on these graphs, the proposed method has a higher TPR and a lower FPR, and the area under the ROC curve is higher than the compared methods. Thus, it can be concluded that the method proposed in this article has a higher average accuracy in the correct classification of melanoma samples. The numerical values related to the tests performed in this paper are given in Table 1.

**Table 1:** Comparing the efficiency of the proposed method with other methods.

Database	Method	MCC	CSI	Accuracy	F-measure	Recall	precision
ISIC-2016	Proposed(ANN + IGWO)	0.9131	0.8636	**97.0976**	**95.5817**	**97.1864**	**94.1755**
	Proposed (CNN Only)	0.7967	0.6867	92.8760	89.5416	92.5461	87.2953
	ANN (LM)	0.8029	0.6953	93.1398	89.8846	92.7105	87.7296
	ANN + GWO	0.8830	0.8171	96.0422	94.0309	96.0263	92.3487
	Nawaz et al.^11^	0.8694	0.7972	95.5145	93.2967	95.6974	91.3485
	InSiNet^12^	0.8920	0.8322	96.3061	94.4544	96.6930	92.6010
ISIC-2017	Proposed (ANN + IGWO)	0.8641	0.7928	**95.1667**	**92.8606**	**96.3503**	**90.2746**
	Proposed (CNN Only)	0.7767	0.6528	92.3333	88.5792	91.3521	86.4683
	ANN (LM)	0.7910	0.6783	92.6667	89.1980	92.5307	86.7776
	ANN + GWO	0.8384	0.7516	94.3333	91.6062	94.8612	89.1716
	Nawaz et al.^11^	0.7966	0.6810	93.1667	89.6725	91.8697	87.8916
	InSiNet^12^	0.8502	0.7716	94.6667	92.1440	95.7159	

Significant values are in [bold].

Based on the results presented in Table 1, the proposed method can diagnose skin cancer with higher efficiency in terms of different criteria. These results show the effectiveness of the proposed strategy in applying it to real world scenarios.

## Conclusion

In this article, a new method for SCD was presented. In the proposed method, the combination of optimization techniques and ANNs is used to diagnose the disease. Also, the combination of Kohonen neural network and GSA is utilized to segment the input images. In this process, the Kohonen neural network creates an initial approximation of the target region in the input images and then the boundaries of these areas are improved by using GSA. This method also uses a CNN model to extract the features of the target region. The proposed solution for diagnosing SC uses an ANN and its configuration and weight vector are determined using the IGWO algorithm. In the proposed IGWO algorithm, a probability vector is used to store the best solutions and move the population based on this model. This probabilistic model is used to increase the convergence speed of the optimization algorithm and can be effective in discovering the global optimum of the problem. The proposed method has been evaluated using the samples of two databases ISIC-2016 and ISIC-2017 and the results have been compared with other methods. Based on the results, the combination of IGWO optimization and ANN in the proposed method can increase the detection accuracy by 2.5% compared to CNN models. On the other hand, the proposed SCD system can detect skin cancer in the ISIC-2016 and ISIC-2017 datasets with an average accuracy of 97.09 and 95.17%, respectively; which is at least 0.5% better than the previous methods.

One of the limitations of the proposed method is that the IGWO algorithm consumes more memory and processing power for training the ANN model, compared to conventional algorithms. Because this algorithm requires the use of a relatively large population to ensure the discovery of the optimal solution. But due to the significant increase in accuracy of the NN by the proposed algorithm, it can be ignored. In future research, the proposed IGWO algorithm can be used to optimize other learning models (such as determining the optimal radius of fuzzy rules in the neural-fuzzy network). Also, in future studies the process of improving the boundaries determined for the target region can be improved by combining the proposed segmentation algorithm with optimization algorithms.

## Data Availability

All data generated or analysed during this study are included in this published article.
